# Magnetite-Amyloid-*β* deteriorates activity and functional organization in an *in vitro* model for Alzheimer’s disease

**DOI:** 10.1038/srep17261

**Published:** 2015-11-26

**Authors:** Sara Teller, Islam Bogachan Tahirbegi, Mònica Mir, Josep Samitier, Jordi Soriano

**Affiliations:** 1 Departament d’Estructura i Constituents de la Matèria, Universitat de Barcelona, Barcelona, E-08028, Spain; 2 Nanobioengineering Group, Institute for Bioengineering of Catalonia (IBEC), Barcelona, E-08028, Spain; 3 Departament d’Electrònica, Universitat de Barcelona, Barcelona, E-08028, Spain; 4 Centro de Investigación Biomédica en Red en Bioingeniería, Biomateriales y Nanomedicina (CIBER-BBN), Barcelona, E-08028, Spain

## Abstract

The understanding of the key mechanisms behind human brain deterioration in Alzheimer’ disease (AD) is a highly active field of research. The most widespread hypothesis considers a cascade of events initiated by amyloid-*β* peptide fibrils that ultimately lead to the formation of the lethal amyloid plaques. Recent studies have shown that other agents, in particular magnetite, can also play a pivotal role. To shed light on the action of magnetite and amyloid-β in the deterioration of neuronal circuits, we investigated their capacity to alter spontaneous activity patterns in cultured neuronal networks. Using a versatile experimental platform that allows the parallel monitoring of several cultures, the activity in controls was compared with the one in cultures dosed with magnetite, amyloid-β and magnetite-amyloid-*β* complex. A prominent degradation in spontaneous activity was observed solely when amyloid-*β* and magnetite acted together. Our work suggests that magnetite nanoparticles have a more prominent role in AD than previously thought, and may bring new insights in the understanding of the damaging action of magnetite-amyloid-β complex. Our experimental system also offers new interesting perspectives to explore key biochemical players in neurological disorders through a controlled, model system manner.

AD is a neurodegenerative disorder characterized by a widespread functional deterioration of the human brain. Among the diverse factors involved in AD pathogenesis, it has been suggested that the high accumulations of amyloid-*β* (A*β*) fibrils that constitute the observed senile plaques^1^,^2^,^3^, as well as the high levels of iron concentration^4^,^5^,^6^, are primary actors. The origin of these abnormal overproductions, their interrelation and actual action on brain’s circuitry are still unsettled questions and a major focus of research.

On the one hand, intermediate states of the final conformation of A*β* plaques, specifically A*β* protofibrils^7^,^8^,^9^, appear to be directly related to neuronal damage. Several studies have indeed demonstrated that these protofibrilar A*β* states alter signal-transduction cascades and cause high neurotoxicity^10^,^11^,^12^. This contrasts with monomeric states of A*β*, which are considered non-toxic.

On the other hand, Fe^2+^, a redoxactive and highly damaging valence state of iron, has been reported in abundant quantities in AD brains^13^,^14^. These abnormal quantities of iron have been linked in turn to magnetite, the only stable iron oxide that contains Fe^2+^ 15,16,17. However, the role of magnetite itself, i.e. either as a natural mechanism for reducing Fe^2+^ toxicity, or as a catalyzer of damage, is still unclear.

The interrelation between A*β*, iron and magnetite has been recently started to be uncovered. Tahirbegi *et al.*^18^ showed that the weak electrostatic interaction between iron and A*β* is stabilized with the crystallization of the magnetite nanoparticle structure, giving rise to a protofibrillar magnetite-A*β* complex (M-A*β*). It was therefore hypothesized that magnetite itself could be non-toxic and even protective against Fe^2+^, but its formation in the presence of A*β* could foster the accumulation and damaging role of the latter.

Despite the advances towards an understanding of key molecular mechanisms in AD, the complexity of the pathology and the inherent difficulty to study the progression of the disorder *in vivo*, have motivated the development of more accessible *in vitro* approaches. Studies using cell cultures have established a connection between fibrillar A*β* plaques and neurotoxic effects^19^,^20^; and in slices from mouse hippocampus it was reported local neuronal damage upon presence of protofibrillar A*β* structures^21^.

In the context of these *in vitro* studies, we here introduce a new experimental paradigm to shed light on the damaging action of magnetite, A*β*, and M-A*β* complex in the functional connectivity of rat cortical cultures. Our neuronal network consists of a set of interconnected aggregates of neurons (clusters) cultured in mm-sized cavities of easy access and manipulation. These *clustered neuronal networks* were introduced before to study the interplay between dynamics and network structure^22^,^23^,^24^, and we have recently exposed their potential to link network functional traits with resilience to damage^25^.

A remarkable feature of clustered networks is their modular activity^25^,^26^, in which clusters fire collectively in small groups forming *dynamical moduli* or *communities*, and that shape what we call the *functional organization* of the network. The coherence within and between communities is tied to the clusters’ interconnectivity, and therefore a loss in coherence can be ascribed to actual changes in the network’s underpinned circuitry due to damage. Our study shows that, of the three chemical actions studied, only M-A*β* complex causes severe network deterioration. This result supports the hypothesis that protofibrilar A*β* structures are stabilized, and its damaging action reinforced, upon presence of magnetite. Our experimental system, together with the derived functional connectivity tools, shape a consolidated *in vitro* model for AD that can be easily extended to investigate other molecular actors of the disorder, and provide a widespread platform for pharmacological studies.

## Results

### Clustered neuronal cultures are suitable platforms to study the effect of amyloid-*β* compounds

**Figure 1:**
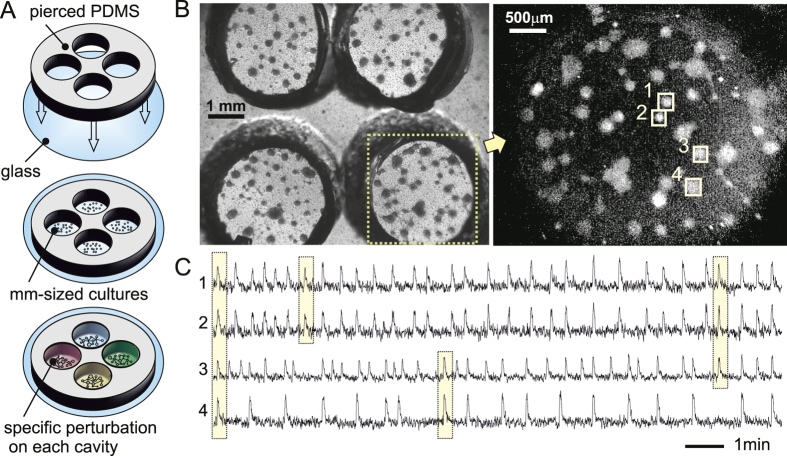
Experiments and data acquisition. (**A**) Sketch of the experimental setup and procedure. A pierced PDMS layer was attached to a glass coverslip to shape a 2 × 2 grid of cavities, each 3.5 mm in diameter and 4 mm deep. Neurons were plated on these cavities forming independent, mm-sized neuronal networks, and cultured in identical conditions. For measurements, one of the cavities was left as control, while the others were dosed with specific chemical agents. (**B**) Left: Bright field image of a typical preparation at *day in vitro* 8, showing the 4 cavities containing the neuronal networks. Dark circular objects are aggregates of neurons (*clusters*) connected to one another through bundles of neurites. Right: Fluorescence image of the bottom-right culture. Firing clusters appear as bright spots on the images. (**C**) Representative fluorescence traces of the 4 clusters highlighted in (**B**), and along 15 min of recording. The yellow boxes illustrate different combinations of co-activations. Clusters #1 and #2 always fire together and shape a strong, persistent community.

We used primary cultures of rat embryonic cortical neurons in our experiments. To study the influence of chemical perturbations on their activity, we developed a simple yet advantageous experimental platform consisting in an array of 2 × 2 cavities pierced on a PDMS layer, each cavity 3.5 mm in diameter and 4 mm deep (see Methods and Fig. 1A). Neurons were seeded with identical nominal density in each cavity, and the entire system cultured as a single unit in a multi-well culture plate to ensure identical development.

The absence of adhesive proteins in the substrate facilitated neuronal aggregation, ultimately giving rise to a set of neuronal assemblies (clusters) connected to one another. These ‘clustered networks’ contained about 30–40 clusters per cavity (Fig. 1B, left), a number that remained stable along development, and exhibited rich spontaneous activity already at *day in vitro* (DIV) 5–6. Although each cavity gave rise to different network designs, their dynamics were qualitatively very similar.

Spontaneous activity was monitored using fluorescence calcium imaging (see Methods). Measurements were carried out at DIV 5–15, which corresponds to the period of highest activity in the networks (about 1–2 firings/min per cluster). Active clusters appeared as bright spots upon activation (Fig. 1B, right), and the monitoring of all the clusters for about 1 h provided the main data for quantifying network activity. Typical fluorescence traces for 4 representative clusters are shown in Fig. 1C. Sharp increases in fluorescence reveal the activation of a cluster, which can fire either spontaneously or as a triggered activation from upstream connected clusters. The example of Fig. 1C also shows that clusters typically fire in groups, forming moduli or communities that are often well preserved along the measurement^25^,^26^.

The novelty of our system is the ability to simultaneously monitor the changes in spontaneous activity in all 4 cavities, and upon administration of a specific chemical agent in each of them. As described in the Methods section, one of the cavities was always left as control, while the others were dosed with either magnetic nanoparticles (M), an A*β* solution, or M-A*β* complex. Data analysis in the context of activity variability, modular organization and functional connectivity provided a quantification of the influence of each chemical agent on network behavior.

### M-A*β* complex degrades spontaneous activity

In a typical experiment we first measured spontaneous activity on each cavity for 30 min, then applied the chemical agents, and measured again for another 30 min (see Methods). The unperturbed, control culture was used to correct for global drifts in activity from the first to the second recording, an activity that either remained the same or increased (by 30% at most).

**Figure 2:**
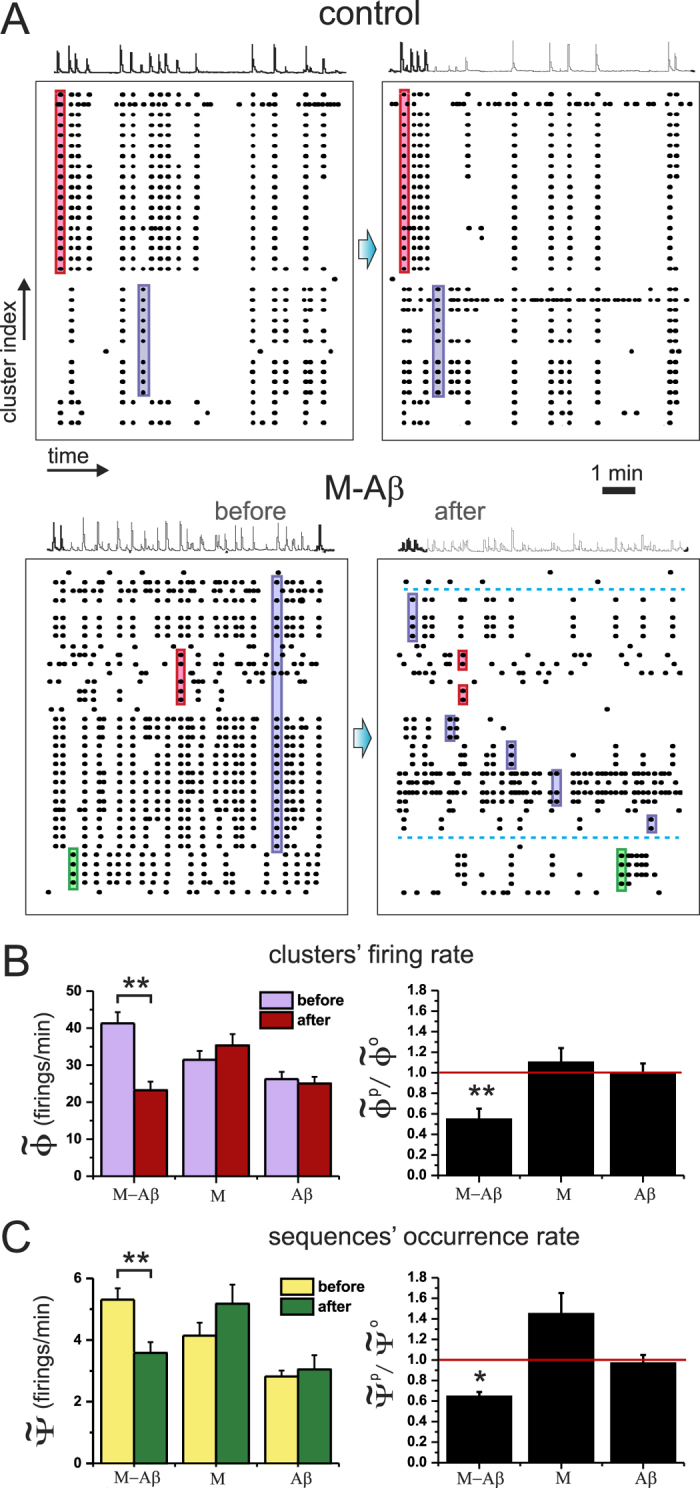
Analysis of the spontaneous activity. (**A**) Representative raster plots of spontaneous activity and average fluorescence signal, comparing the behavior of the clustered neuronal networks in control conditions (top) and under perturbation with M-A*β* complex (bottom). The left panels show the first 10 min of recording before perturbation, while the right panels show the last 10 min of recording upon perturbation. For the control case, the network activity and the structure of the different firing sequences (outlined boxes) are preserved, while for the M-A*β* case both traits degrade, with a rupture of the biggest sequence in sub-sequences and the silencing of clusters (dotted horizontal lines). (**B**) Variation of the mean clusters’ firing rate 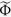 upon perturbation, and comparing the action of M, A*β* and M-A*β*. The left panel shows the values before and after application, and corrected by the control measurement; the right panel shows the corresponding ratios. (**C**) Complementary analysis considering the sequences’ occurrence rate 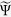. Each dataset is an average over 15 experimental realizations, and is represented as mean +/− standard error of the mean. (*p < 0.05, **p < 0.005, Student’s t-test.) The red horizontal line is a guide to the eye for the control condition.

The raster plots in Fig. 2A exemplify the collected data and their analysis. The left and right panels show, respectively, the network activity before and after the chemical intervention. The control case effectively consisted in two identical, consecutive measurements, and its raster plots indeed exhibit nearly the same features. The network is characterized by rich spontaneous activity with the existence of two distinct groups of coherent activations (*firing sequences*, highlighted as color boxes). Both network activity and the pattern of the repeating firing sequences are well preserved along the two recordings.

The stability of the control measurement contrasts with the behavior of the M-A*β* cavity. Spontaneous activity is rich before chemical application, with 3 distinct repeated firing sequences, but substantially decays after dosage, with a rupture of the pattern of the firing sequences in smaller subsequences. For instance, a firing sequence highlighted in blue on the left panel —and that encompassed the majority of the clusters— broke off into 4 new, uncorrelated subsequences. One can also observe that some clusters became completely silent (dotted lines), while others boosted in activity.

We extended the analysis to 15 experimental realizations, and compared the behavior upon M, A*β* and M-A*β* action. A first evaluation of the changes in the dynamics of these networks was carried out by computing the average clusters’ firing rate 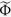 (see Methods). As shown in the left panel of Fig. 2B, 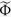 significantly decayed by about a factor 2 upon M-A*β* action (p-value *p* = 8 × 10^−6^), while it remained unaltered within statistical error for both M and A*β* perturbations. To compare the variability in behavior between chemical actions, we computed the ratio between the spontaneous activity after and before perturbation, 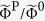. As shown in the right panel of Fig. 2B, activity significantly dropped upon M-A*β* action when compared with either the M or A*β* cases (*p* = 6 × 10^−5^ and *p* = 4 × 10^−4^, respectively). No significant differences were observed between M and A*β* (*p* = 0.3).

Since clusters do not fire independently but rather within communities, in a second analysis we computed the *sequences occurrence rate* 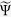, i.e. treating as a single activity episode all those clusters that fired together within a given time window (see Methods). The results comparing 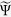 before and after perturbation are shown in the left panel of Fig. 2C and depict a significant fall in the number of observed sequences upon M-A*β* action (*p* = 4 × 10^−4^). Again, for M and A*β* the changes in activity are not significant (*p* = 0.2 and *p* = 0.9, respectively). The comparison between the ratios of the different actions (Fig. 2C, right panel) reveals a significantly lower sequences’ occurrence rate between M-A*β* and the other actions (*p* = 3 × 10^−5^ in both cases), but not between M and A*β* (*p* = 0.7).

**Figure 3:**
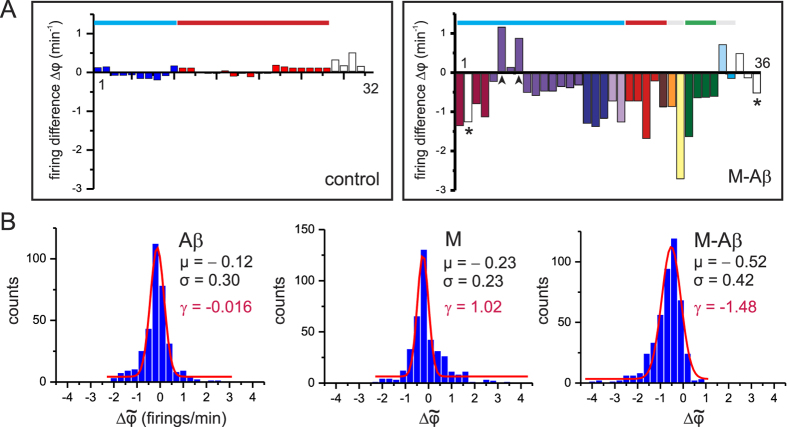
Cluster’s activity and network coherence. (**A**) Difference in cluster’s firing rate between the perturbed (*φ*^P^) and the unperturbed (*φ*^0^) activities, and comparing a control cavity (left, 32 clusters) with one targeted with M-A*β* complex (right, 36 clusters). Data corresponds to the experiments shown in Fig. 2A. Each bar represents a cluster of the network. Clusters are color coded and ordered in the horizontal axis according to their participation in a co-activated group. White bars depict clusters that fire independently. Bars marked with asterisks indicate the clusters that became silent after perturbation, and the ones marked with arrowheads highlight those that boosted in activity. The top horizontal color boxes show the structure of co-activations before perturbation, and color coded according to the sequences shown in Fig. 2A. Grey boxes are sequences that were not indicated in Fig. 2A. (**B**) Distributions of the normalized firing rate differences 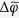 for 15 experimental realizations upon action of the different chemical agents. The red curve shows a Gaussian fit to the distributions, with mean *μ* and standard deviation *σ*. The value *γ* provides the skewness of the distributions.

To deepen in the understanding of the M-A*β* damage on network dynamics we analyzed in detail the representative experiment of Fig. 2A, and computed the difference in spontaneous activity before and after perturbation for each individual cluster, Δ*φ* = *φ*^P^ − *φ*^0^ (see Methods). As shown in Fig. 3A, clusters in the control case slightly varied in activity due to the natural fluctuations in such a biological system, but the overall population activity along the two measurements remained stable, with 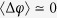. The structure of clusters’ co-activations was characterized by the two major communities outlined in the raster plot of Fig. 2A together with few clusters that fired independently. This dynamical organization was the same for both measurements, and illustrates the stability of the clusters’ coherence in control conditions.

For the M-A*β* case, however, there was a remarkable drop in the activity of most of the clusters together with a rupture of the initial co-activation patterns. The biggest community of coherent clusters indeed divided into 5 smaller groups upon perturbation. Some clusters also became totally silent (marked with asterisks) while others boosted in activity (arrowheads). Although this particular experiment exhibited two clusters with boosted activity, such a feature was in general rare.

We extended this analysis to the M and A*β* cases, and included all the 15 experimental realizations. Figure 3B shows the distribution of normalized clusters’ activity differences 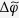 (see Methods) for all the data. For the A*β* case, all clusters were active along the recording and their activity varied moderately, with an overall symmetric distribution centered at 0 (mean 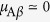, skewness 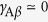). The M case shared similar characteristics except for a higher presence of boosted activations, as reflected by its right–skewed distribution (*γ*_M_ = 1.02). Such a potentiation of activity by the magnetic nanoparticles is an interesting observation that needs further studies to be understood. Conversely, the M-A*β* case exhibited a prominent widespread loss in clusters’ activity (often caused by clusters that became silent), with a remarkable shift of the distribution towards negative values (*μ*_M−A*β*_ = −0.52, *γ*_M−A*β*_ = −1.48), and the almost absent presence of clusters with boosted activity.

These results suggest that M-A*β* complex damaged the connectivity between clusters, the clusters themselves, or both. Such an action caused a deterioration of the individual clusters’ activity and the overall network collective behavior.

### M-A*β* complex disrupts the structure of network communities

**Figure 4:**
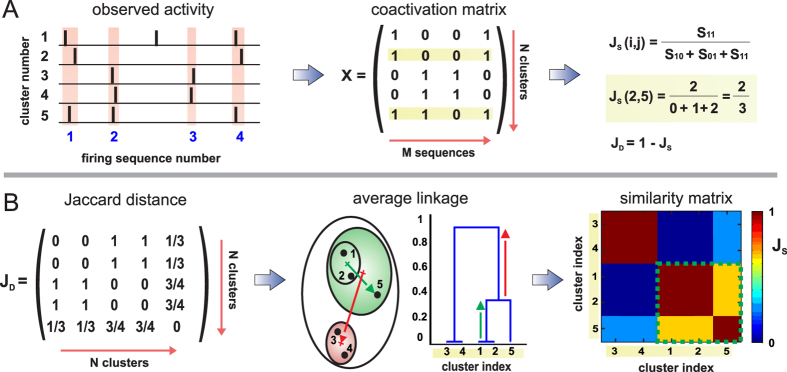
Communities’ analysis methodology. (**A**) Schematic representation of spontaneous activity traces for 5 clusters. All those clusters that fire concurrently in a short time window define a sequence (pink bars). Clusters that fire independently are discarded. Sequences shape the *X* matrix, where each column corresponds to a sequence, and each row represents the activity history of a cluster. Two or more clusters that share a similar history are more likely bound and would constitute a community. The degree of similarity between all pairs of clusters (*i*, *j*) is established through the Jaccard’s similarity measure *J*_*S*_(*i*, *j*), from which the Jaccard’s matrix distance *J*_*D*_ = 1 − *J*_*S*_ is determined. Clusters #1 and #2 are identical in history and provide *J*_*S*_(1, 2) = 1, but both are also similar to #5 (yellow bands), with *J*_*S*_(1, 5) = *J*_*S*_(2, 5) = 2/3. (**B**) *J*_*D*_ is a symmetric matrix that reflects the relative closeness of all pairs of clusters, which can be sketched as spatial groups or in the form of a dendrogram. Clusters #3 and #4 have identical histories and form a unique community. Clusters #1 and #2 also shape a community, but they are sufficiently close to cluster #5 to constitute together a higher, more representative group. The number of communities is formally set by selecting a threshold in the dendrogram. Any threshold along the red arrow would maintain two communities. Once a threshold is set, the similarity matrix is ordered to visually highlight the communities in the network.

**Figure 5:**
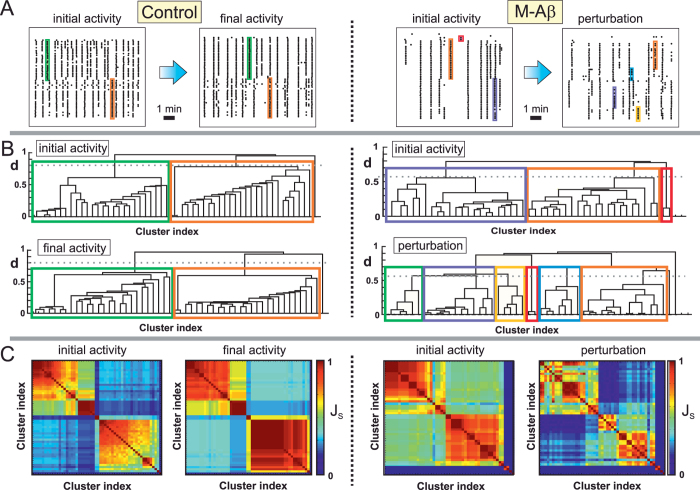
Communities analysis. (**A**) Raster plots of spontaneous activity for a control (left) and a M-A*β* perturbation (right) experiments. Outline boxes depict the most important firing sequences and that shape specific communities. (**B**) Corresponding dendograms and Jaccard similarity matrices. For the control case, the dendrograms compare the network’s communities (color boxes, set with a threshold *d*_th_ = 0.80) in two consecutive measurements, which exhibit similar traits and the maintenance of the two main communities. For the M-A*β* case, the dendrograms compare the standard and the perturbed measurement (*d*_th_ = 0.58), and reflect the rupture of the two main communities and the formation of smaller ones. Boxes are color coded according to the firing sequences in (**A**). (**C**) Corresponding Jaccard similarity plots drawn using the clusters’ indexes provided by the dendrogram, highlighting the deterioration of the network upon M-A*β* action.

We illustrate the damage in the collective dynamics of the network by considering another representative experiment in which all clusters displayed activity after M-A*β* perturbation. An overview of the methodology that we used is shown in Fig. 4, and all the details are provided in the Methods section. Figure 5A shows the raster plots of the analyzed experiment, which exhibits the usual stability for the control case and the break down of clusters’ coherence upon M-A*β* application.

Figure 5B shows the identification of the network communities through the analysis of hierarchical trees or dendrograms, which classify and order the clusters according to their similarity in activity along the recording. By setting a threshold in the dendrogram (dotted horizontal lines) the most representative communities in the network can be established (see Methods and Supplementary Figure 1). For the control case we obtained 2 characteristic communities that encompass around 15–20 clusters each. These communities are well preserved along the two consecutive recordings, although with variations in their internal structure due to fluctuations in clusters’ dynamics. For the M-A*β* case, however, the structure of communities markedly changed, with 3 main communities separating into 6.

Figure 5C shows an alternative representation of the communities in the form of the similarity matrices *J*_*S*_. The control experiment retained the main structure of the communities despite fluctuations, while for the M-A*β* case the reorganization of the network in smaller communities was well manifested.

**Figure 6:**
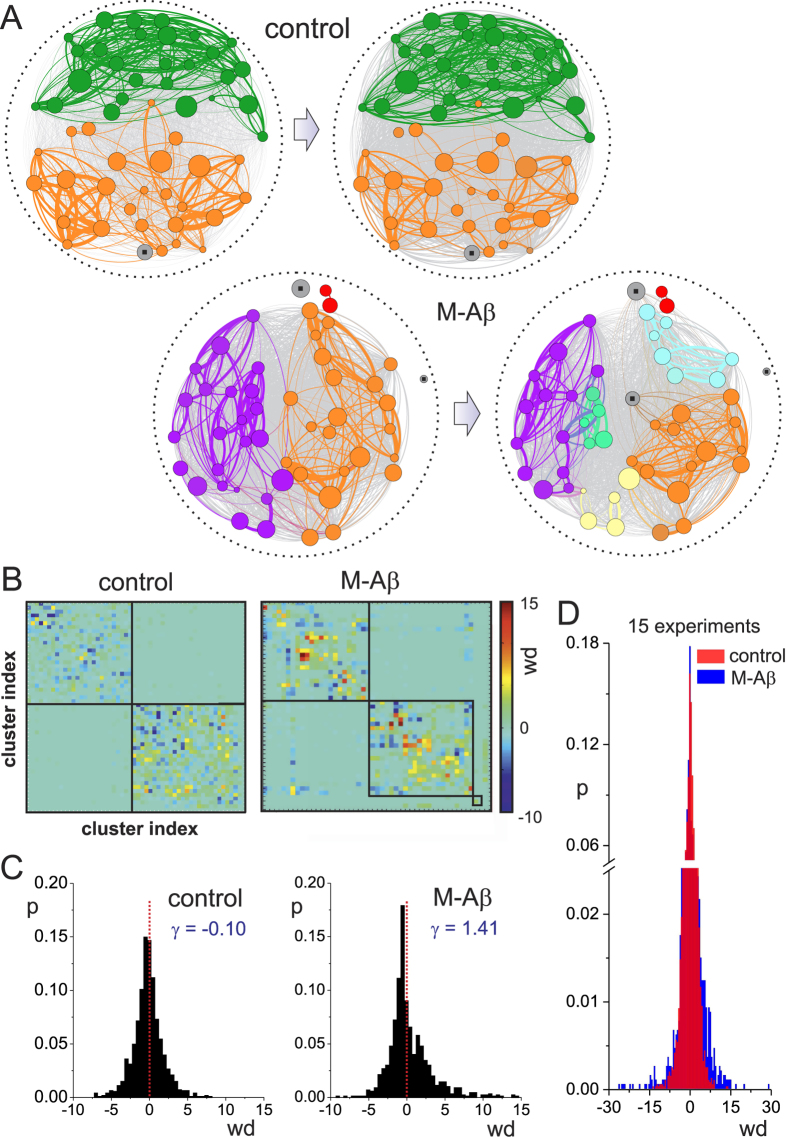
Functional connectivity and damage. (**A**) Functional networks for control and M-A*β* experiments. Gray links show all functional connections, and color links correspond to the top functional connections (z - score > 1.95, 500 surrogates). The thickness of a link is proportional to its weight. The direction of the links is not shown for clarity. The control network reflects the stability of the network in two consecutive measurements. The M-A*β* network exhibits strong connectivity changes and reorganization of the network moduli, which are color coded according to the hierarchical tree information of Fig. 5. Grey clusters with a square in their center are those that fired independently or that participated equally in different communities. (**B**) Matrices of weight differences *wd* of the functional links. The clusters’ index order is the same as in Fig. 5, and corresponds to the unperturbed condition. The square boxes outline the moduli before perturbation. (**C**) Corresponding distributions of weight differences for the control and M-A*β* experiments. *γ* indicates the skewness of the distributions. The red vertical line is a guide to the eye to show the symmetry of the distribution in the control case. (**D**) Comparison of the distributions including all 15 experimental realizations. The M-A*β* distribution is broader and contains both strongly weakened and strongly strengthened functional links.

We combined the analysis of the communities’ structure with the functional connectivity of the network to better quantify the changes in the coupling between clusters upon perturbation. As described in Methods, functional maps were obtained by analyzing the time delays in the clusters’ activations. In this construction, the shorter the time delay the stronger the directed functional bond (*weight*) between clusters. Figure 6A depicts the functional maps for the control and M-A*β* experiments. Two levels of representation are shown. In a first one, all functional connections between clusters are drawn as gray links, effectively shaping a homogeneous area that evinces the widespread clusters’ functional interconnectivity. In a second one, only the top functional connections (z - score > 1.95, 500 surrogates) are shown, with clusters and their connections color coded according to their participation in the above inferred communities. The thicker a connection is, the higher is the weight of the functional bond. For the control case, we observed that the overall structure of the network was well preserved along the two recordings, with small variations in the functional links that reflect the fluctuations observed in the dendrograms. This maintenance of the network features was also observed in the M and A*β* cases (see Supplementary Figure 2). For the M-A*β* case, the rupture of the 3 initial communities into 6 smaller ones was clear, and the clusters that remained in a given community experienced important variations in functional connectivity. However, a number of the strongest links were preserved, hinting at the maintenance of some sort of internal organization in the network. Another example of a M-A*β* perturbation is provided in the Supplementary Figure 2, and corresponds to the data shown in Figs 2A and 3A.

To quantify in more detail the changes in the functional connectivity we computed the weights’ differences *wd* of the functional links between the perturbed condition and the initial one. As shown in Fig. 6B, the weights fluctuated in the control case, but variations occurred within the communities and with few extreme values. For the M-A*β* case, however, extreme variations were abundant, and with a large presence of negative values outside the two main original communities. This broad network weakening ultimately caused the emergence of the smaller communities and the overall degradation of network activity. Such a deterioration can be also pictured by plotting the distribution of *wd* values. As shown in Fig. 6C, the control case led to a symmetric, narrow distribution centered at zero (skewness *γ*_C_ = −0.10). Conversely, the M-A*β* displayed an asymmetric distribution with two main features. First, the peak of the distribution appeared at negative values, which signatures an overall network weakening; and second there was an emergence of extreme positive differences (*γ*_M−A*β*_ = 1.41) that possibly reflect the activation of network mechanisms to stop activity degradation by reinforcing specific links. Such a behavior is also maintained across the 15 experimental realizations studied (Fig. 6D). However, we must note that network response to damage and functional reorganization is complex. Although inter–moduli connectivity conformed most of the negative weight differences, intra–moduli connectivity featured a similar number of positive and negative differences.

**Figure 7:**
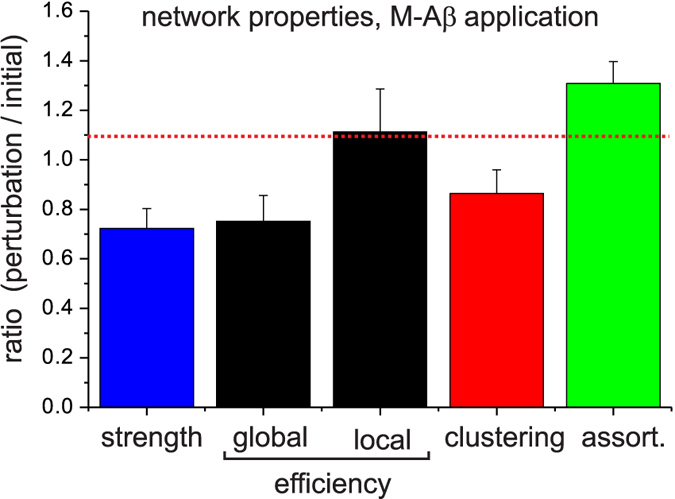
Changes in network topology upon M-A*β* perturbation. Ratio of five topological descriptors, namely network strength, global and local efficiencies, clustering coefficient, and assortativity, after and before the application of M-A*β*. The descriptors are computed from the directed and weighted functional connectivity graphs, and averaged over 15 network realizations. Error bars indicate mean +/− standard error of the mean. The red horizontal line is a guide to the eye for the control condition.

To complete the analysis of the functional organization of the network upon M-A*β* action, we also analyzed high–order topological features such as the efficiency, clustering, and assortativity (see Methods). The results are summarized in Fig. 7, and are presented as the ratio between the condition of the network after M-A*β* application and before it. The *strength*, understood as the sum of the weights of all links, substantially decayed after perturbation and reflects the widespread loss of connectivity density seen in the functional maps. The *global efficiency*, which captures the performance of the network as a whole, also substantially decreased, and indicates that the perturbation affected the global integration among the communities. This is supported by the increase of the *local efficiency*, overall pinpointing a scenario where the information flow shifted towards local areas. The fall of the *clustering coefficient*, on the other hand, indicated the emergence of a sparser connectivity, i.e. a switch towards a more random network.

The combination of a decreased global efficiency with an increased local one strengthens the observation that M-A*β* action caused the partition of the biggest moduli. This community disruption is also reflected by an increase of the *assortativity*, the tendency of the nodes to connect with others of similar degree. We must note that modular networks are in general assortative, and that the clustered networks already exhibit this property^25^. Hence, the observed increase in assortativity indicates that the networks maintain the modular organization despite deterioration, a feature that in turn favors the preservation of activity, though local, in the system (see e.g. Fig. 2A).

### Clustered networks exhibit higher resistance to M-A*β* damage compared to homogeneous ones

As a final analysis, we studied the sensitivity of extremely different neuronal network configurations to damage. We compared clustered neuronal networks with standard, homogeneous preparations in which neurons covered uniformly the glass substrate (see Methods). The rationale behind this comparison is the existence of the assortative traits outlined above, a topological feature that has been also associated to network resistance to damage^27^. It was shown that while the assortative, clustered networks were resilient to the weakening of connections, the weakly dissassortative, homogeneous ones were not^25^.

**Figure 8:**
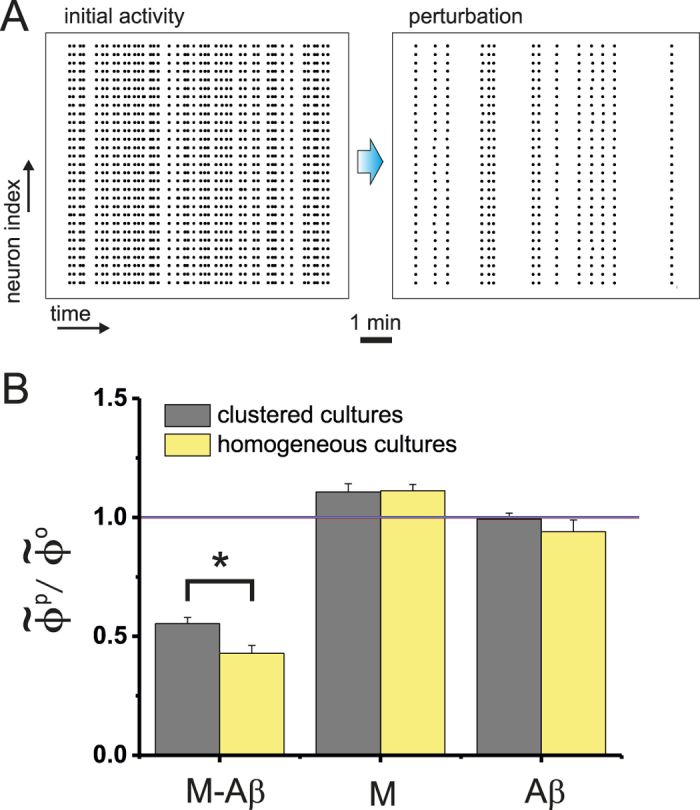
Homogeneous cultures. (**A**) Raster plots of spontaneous activity before (left) and after (right) application of M-A*β* complex. Spontaneous activity fell by 65% on average. (**B**) Ratio of the normalized firing rate for homogeneous (averaged over 5 experiments with the respective standard error of the mean) and clustered cultures (15 experiments), and comparing the behavior of the two kind of cultures for the M, A*β* and M-A*β* perturbations. Both culture types behaved similarly except for the M-A*β* condition, where the homogeneous cultures exhibited a significantly higher decay in activity. (*p < 0.05, Kolmogorov-Smirnov test.)

Spontaneous activity in homogeneous cultures is characterized by episodes of collective dynamics (termed network bursts) combined with silent intervals. Figure 8A shows a typical raster plot of activity before and after application of M-A*β*. Network coherence was preserved upon perturbation, but the frequency and regularity of bursting episodes were substantially reduced. The number of bursting episodes kept decreasing as the damage progressed until activity ceased. This behavior contrasted with the one observed in clustered networks, where whole network activity switched to a modular yet highly rich one that lasted for long time. In general we observed that homogeneous cultures became silent much earlier than clustered ones, a result that provides a qualitative evidence for a higher resistance of the latter to damage.

To quantify the differences between the two culture types, we analyzed the ratio in the normalized spontaneous activity of either neurons (for the homogeneous preparations) or clusters, 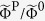, and compared the action of M-A*β* with the other perturbations. As shown in Fig. 8B, homogeneous cultures exhibited a significantly higher drop in spontaneous activity than clustered ones (*p* = 0.04), while for the M and A*β* cases both culture types behaved similarly within statistical error.

## Discussion

The A*β* peptide is a central compound in the study of Alzheimer’s disease. Its implication seems clear from the large quantities of amyloid plaques observed in histological samples from AD patients^28^. However, the A*β* effects on neurodegeneration are a subject of debate^29^,^30^. The reported studies range from considering this peptide primarily responsible of AD (amyloid cascade hypothesis^31^) to dispute its direct relation with the disease^2^,^32^, with new scenarios embracing the idea that A*β* is involved in AD as a secondary player^29^,^30^. The amyloid hypothesis suggests that the deposition of A*β* peptides initiates a cascade of events that ultimately cause widespread neuronal damage. Diverse studies indeed support the link between A*β* plaques and cell membrane damage^33^,^34^, ion channels dysfunction^35^, and neurotoxicity through the generation of reactive oxygen species^36^. However, the lack of clinical success in drugs targeting fibrillar A*β* has evidenced the need for alternative explanations where A*β* is not the principal player^37^. This is supported by studies concluding that fibrillar A*β* deposits in the cortex of AD brains do not correlate with the presence of neurofibrillar tangles and neuronal mortality^38^, and that neurodegeneration may occur before the deposition of A*β* fibrils^32^. Hence, an emerging hypothesis suggests that the Tau protein is the key player in the disorder, with A*β* a secondary actor^29^,^31^. Additionally, another hypothesis points towards magnetite and other metallic nanoparticles^39^, abundant in AD brains and that could catalyze the formation of reactive species^40^.

Given the diverse hypothesis for AD, our work aimed at introducing a new experimental platform and analysis tools for understanding the role of the compounds that seem pivotal in neuronal damage. We centered our efforts in the combined effect of magnetite and A*β*. Our results indicate that A*β* manifests a damaging role only when acting in synergy with magnetite, which possibly acts as a stabilizer for a protofibril M-A*β* complex. Although other studies have shown that A*β* itself induces apoptosis in neuronal cultures similar to ours^41^, in the time frame and conditions of our study we could not find evidences for neuronal damage due to the action of either A*β* or magnetite. It may occur that long exposures to these agents induce neuronal damage and broad alterations in network function, but our results clearly show the aggressiveness of M-A*β*, which acts quickly and broadly.

The paramount result that evince the M-A*β* damage is the combined loss of neuronal activity and the deterioration of network’s functional organization. This was quantified by an average fall of the spontaneous activity, a rupture of the dynamic communities, and widespread changes in the functional connectivity of the culture. Neuronal apoptosis was reflected in our networks by the gradually higher presence of silent clusters. However, network functional deterioration occurred much earlier than actual neuronal death, and suggests that M-A*β* acts first on connections and, at longer stages, on the cells themselves. Despite the degree of damage, the partition of the network into smaller communities has two interesting features. First, the new moduli are constituted by clusters that are spatially close (see e.g. Fig. 6A), and reveals that clusters tend to connect to their nearest neighbors rather than establishing long–range paths. And second, the intra-modular organization is overall preserved despite fluctuations in weight differences, picturing a network with a core of strongly connected nodes. Altogether, the clustered networks effectively configure a *network of networks*, a hierarchical organization that may explain their higher resistance to damage as compared to homogeneous networks.

Clustered networks upon M-A*β* action switch from a global dynamics to a local one and, as far as few modules are active, the network maintains some degree of operation that can help activating response mechanisms. This switching scenario has been also observed in AD^42^,^43^ and mild cognitive impairment^44^. Structural and functional neuroimaging techniques revealed that the first clinical manifestations of AD were associated with a loss of the integration capacity between brain regions, while the segregation, i.e. the reinforcement within neuronal modules, increased^45^,^46^. In this direction, an enlightening result of our study is the capacity of the clustered networks to respond to damage. Indeed, the functional connectivity analysis pinpoints a strong reorganization, with the reinforcement of existing functional paths and the formation of new ones. This behavior shows the intrinsic ability of the network to compensate or restore for damaged function. We conjecture that this plasticity provides mechanisms for response and adaptation against external attacks. Such a fast response is a well known observation in brain recordings^47^. For instance, along the intermediate stages of AD there have been observed episodes of partial recovery of cognitive tasks and memory^48^,^49^, and that possibly reflect broad neuronal circuit response to damage.

Our work provides an innovative and versatile tool to unveil the action of molecular agents in network activity and function. We believe that it is a unique experimental model system for neurodegeneration that may help uncovering universal processes for functional reorganization upon damage. Our system can not only help understanding the role of magnetite, A*β* or M-A*β* complex in Alzheimer’s, but can also help addressing the role of other pivotal players such as the Tau protein. It can also help developing innovative therapeutics for AD. Indeed, given the importance of the combined effect of magnetite and A*β*, it has been suggested to use transcranial magnetic stimulation to target magnetite accumulation, with promising advances. Magnetic stimulation could be incorporated in our experimental system given its accessibility and easy manipulation, and could help exploring in a control manner the benefits of such an action. Also, our system could be useful to test specific drugs at a network level, providing a valuable tool for pharmacological studies focused on AD and other disorders.

## Methods

### Clustered neuronal networks

All experimental procedures were approved by the Ethical Committee for Animal Experimentation of the University of Barcelona, under order DMAH-5461, and in accordance to the regulations of the Generalitat de Catalunya (Spain).

Cortical neurons from E18-19 Sprague-Dawley rat embryos were used in all the cultures, following the procedures described in ref. 25 with minor modifications. Pregnant rats were killed with CO_2_ and the embryonic brains swiftly dissected in ice–cold L–15 medium. The extracted cortices were dissociated by repeated pipetting, and neurons finally seeded onto 13 mm glass coverslips (#1 Marienfeld-Superior) that incorporated 4 circular cavities in a poly-dimethylsiloxane (PDMS) mold. Cavities were 3.5 mm in diameter, 4 mm deep, and were placed forming a 2 × 2 grid with a typical separation of 1 mm. Neurons in each cavity aggregated within days to constitute a network of neuronal islands (*clusters*) connected to one anther.

About 20 wells containing the PMDS-glass structures were simultaneously prepared in each dissection to ensure that the derived networks were as similar as possible. Wells were incubated at 37 °C, 5% CO_2_ and 95% humidity in Plating Medium up to *day in vitro* (DIV) 5, with a complete medium replacement at DIV 3 for refreshment and to eliminate cells debris. Cultures were next treated with 0.5% FUDR and Uridine to limit glial cell division, and from DIV 8 onwards cultured in Final Medium (enriched MEM with 10% HS), with a periodic fluid replacement every three days.

### Experimental setup

Neuronal activity was monitored through fluorescence calcium imaging and using the cell-permeant fluorescence probe fluo-4-AM, as described before^25^,^50^. Each recording consisted in the monitoring of activity in a culture well, i.e. the PDMS-glass structure that contained the 4 neuronal networks within the cavities. Prior measurements, the culture was incubated in recording solution (RS) for 25 min in the presence of 1 *μ*g/ml of Fluo-4. The culture was next transfered to a clean chamber with fresh RS, and left in darkness for 5 min for stabilization.

The well was observed on a Zeiss Axiovert inverted microscope equipped with a high–speed CMOS camera (Hamamatsu Orca Flash 2.8). Neuronal activity was recorded at intervals of 20 ms using the dedicated software Hokawo 2.5. Individual frames were acquired as 8–bit grey–scale images, a size of 960 × 720 (width × height) pixels, and covering a field of view of 8.2 × 6.1 mm^2^ that included the 4 cavities. All experiments were carried out at room temperature.

### Experimental procedure

Spontaneous activity in the 4 cavities was first recorded for 30 min. Next, the RS solution was removed from the recording chamber except in the cavities, effectively establishing 4 completely independent containers filled with RS. One of these 4 cavities was always left as control and remained unperturbed, while the others were doped with 2 *μ*l of the chemical agents of our interest, namely magnetic nanoparticles (M), Amyloid*β* solution (A*β*), or Magnetite-Amyloid*β* complex (M-A*β*). The agents were thoroughly stirred prior use and were delivered through a micropipette. The cavities were next left in darkness for 10 min for the compounds to dissolve and take effect, and spontaneous activity resumed for another 30 min. Cultures were discarded at the end of the recording, and images processed for analysis. Recordings were intentionally reduced to 1 h in order to ensure that the networks did not change in behavior due to photo-damage or other effects^25^.

### Inhibitory synapses

The neuronal cultures contained both excitatory and inhibitory connections. To rule out the effects of inhibition in the analysis of networks’ dynamics, GABA_A_ receptors in inhibitory synapses were blocked with 40 *μ*M of the antagonist bicuculine methiodide (Sigma). The antagonist was applied 5 min before the actual recordings for the drug to take effect.

### Amyloid*β* and M-A*β* complex preparation

The synthesis of magnetite-A*β*42 complex was carried out by mixing 12.5 *μ*mol iron (III) chloride hexahydrate (FeCl_3_-6H_2_O) (Sigma) and 6.25 *μ*mol Iron (II) chloride tetrahydrate (FeCl_2_-4H_2_O) (Sigma) in 10 *μ*l of DDW and added to 0.04 *μ*mol of lyophilized A*β*42. The pH was adjusted afterwards to physiological conditions with 2.6 *μ*l of 7.2 M ammonium hydroxide (Sigma). The solution was sonicated at 37 °C for 30 min to minimize aggregation and self-fibrilization of the A*β*42^51^,^52^. To remove side products from the magnetite-A*β*42 complex, the solution was washed with consecutive cleaning steps with 1 ml ethanol and 1 ml DDW. Between washing steps, unattached amyloid and other side products were separated from the magnetite-A*β*42 complex using a neodymium magnet. The sample was next mixed with 1 ml ethanol and centrifuged at 2400 rpm for 2 min. The liquid was next removed and the sample was kept redissolved in RS for its use in the experiments. The final concentrations were 12.5 mM for Fe^3+^, 6.25 mM for Fe^2+^, and 10 *μ*M for the A*β*42 peptide. These values are similar to the ones reported in brains of AD patients^53^,^54^.

### Data analysis

Each cluster was considered as a region of interest (ROI) from which the trace of fluorescence intensity was extracted. This data was next analyzed to obtain the *onset times* of activation of each cluster. Since the fluorescence amplitude of a cluster ramped up during a firing event, onset times were determined by searching those occurrences in which the amplitude of the fluorescence signal and its derivative were concurrently high, and using a convenient threshold to discern actual firings from the background signal^25^.

### Clusters’ firing rate

As a first measure of spontaneous activity, we considered the clusters as independent units, and computed the firing rate *φ*_*i*_ of each cluster *i* as the number of observed firings per unit time. The difference in individual clusters’ activity after (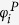) and before perturbation (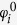) was given by 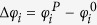. To provide a quantity that represents the average activity in a cavity, we introduced the mean clusters’ firing rate as 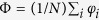, where *N* is the number of clusters, and used Φ^0^ and Φ^*P*^ to denote the activity before and after perturbation, respectively.

Changes in the temperature of the well or medium evaporation can cause variations in the spontaneous activity of all cavities from the first to the second recording. Such a drift is an artifact that masks actual chemical damage, and is quantified as the ratio 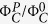, with 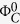 and 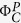 the average activity in the control cavity along the first and second recording, respectively. Of the 15 experiments studied, this ratio ranged between 0.9 and 1.2.

Drift correction for the perturbed cavities in the well was carried out as follows. For the individual clusters’ activity difference we introduced the quantity 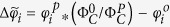; and for average activity we considered 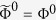 and 
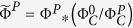
.

### Firing sequences

A firing sequence is defined here as the concatenated activation of two or more clusters within a window of 200 ms. This time was also set as a cut-off to separate two sequences. Sequences typically lasted 100 ms, encompassed about 5–15 clusters, and were separated by about 1–10 s. By construction, clusters that fired independently were excluded from this analysis. Although some of these isolated clusters could fire randomly within the time window of another sequence and be part of it, such events of accidental participation were rare.

### Functional connectivity

Following Teller *et al.*^25^, a directed and weighted functional graph was constructed to provide a measure of the coupling strength between clusters during activity. Two clusters were functionality linked when they participated in a given sequence. The weight of the link was next established as a decaying function of their time delay in activation. The analysis was extended to all observed sequences, and the final value of the functional link *w*_*ij*_ between clusters (*i*, *j*) was finally established as the sum of all computed weights.

A random model 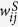 that preserved the firings of each cluster was constructed to evaluate the significance of each functional link, as well as to normalize the weights for comparing different experimental conditions. A z-score was then introduced, defined as


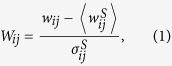


where 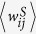 is the mean weight for 500 surrogates of the random model, and 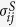 the corresponding standard deviation. The matrix *W* procured the normalized weights between all the functionally connected clusters in the network. Those links with a high *W* score appeared frequently and therefore their clusters were strongly connected. At the other extreme, negative *W* scores reflected those links that were less connected than in a random configuration and therefore disregarded. Those *W* scores above the 95% confidence interval were the ones finally used to compare the functional connectivity before and after chemical action.

### Sequences’ occurrence rate

The sequences’ activity Ψ was introduced as a second measure of spontaneous activity in the network, and was given by the total number of identified firing sequences *M* per unit time, and used Ψ^0^ and Ψ^*P*^ to denote, respectively, the initial measurement and the perturbed one. Drift correction was set as 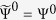 and 
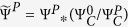
, with 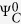 and 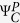 the sequences’ occurrence rate of the control cavity.

### Sequences’ similarity analysis

Firing sequences that occurred frequently reflected the existence of distinct communities within the network. These communities were rendered using the hierarchical clustering algorithms described in refs 55,56. To proceed, a coactivation matrix *X* containing *N* × *M* elements was initially considered, where *N* accounted for the number of variables (neuronal clusters) and *M* for the number of distinct observations (firing sequences).

Once all the sequences in a given measurement were established, the elements were set to *X*_*ij*_ = 1 if cluster *i* had participated in the sequence *j*, and *X*_*ij*_ = 0 otherwise. At the end of the construction, the rows of the matrix *X* (with length *M*) reflected the activity history of a given cluster. A silent cluster contained all the elements of its row equal to zero.

*Jaccard metrics*^56^,^57^ was next used to calculate the pairwise similarity between the activity history of the clusters in the network. If *A* and *B* are any two rows of the matrix *X*, the *Jaccard similarity J*(*A*, *B*) provides a score that indicates the similarity in the history of *A* and *B*. It was calculated as the number of concurrent activations in the two clusters respect to all the occurrences where at least one of the clusters had fired. If *A* and *B* had no common activations (i.e. *A* and *B* have all elements as 0), then *J*(*A*, *B*) = 0; and if *A* = *B* (all elements equal) then *J*(*A*, *B*) = 1.

The matrix *J* was finally ordered to group those clusters with similar high scoring, which highlighted those groups of clusters that formed distinct communities in the network. The matrix index was provided through the analysis of the hierarchical tree (dendrogram) on the *Jaccard distance D* = 1 − *J*, which was evaluated using the average linkage method (Statistical Toolbox package, Matlab).

### Communities analysis

To mathematically identify which clusters shaped a particular community, a threshold had to be established in the hierarchical tree. However, the actual values of the Jaccard distance were different across measurements, and therefore a normalization had to be introduced to consistently compare changes in the communities across measurements. Normalization was carried out by using a z-score respect to a null model that consisted on a series of 500 surrogates of *X*, in which its rows were randomly reshuffled. We note that this reshuffling preserved the number of firings of each cluster. Each surrogate provided a corresponding Jaccard distance matrix *S*. The matrix of z-score values *Z* was next computed as


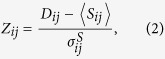


where 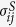 is the standard deviation of the family of surrogate values *S*_*ij*_. 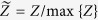 was finally computed to provide Jaccard distances 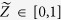.

The analysis of 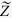 in the form of a dendrogram gave a visual representation of the organization of the clusters into communities. To provide representative communities in the network and compare their structure before and after perturbation, a threshold *d* in this dendrogram had to be set. The larger the threshold, the smaller the number of communities. The optimum value for the threshold was obtained through the computation of the Variation of Information (see below), and provided the minimum yet significant number of communities in the system that was not the trivial case of a single community.

### Variation of Information and optimum threshold for community detection

The Variation of Information VI is an information-theoretic measure that compares any two partitions *X* = {*x*_1_, *x*_2_, ... *x*_*N*_} and *Y* = {*y*_1_, *y*_2_, ... *y*_*N*_}, with *N* the number of elements. In our case, *N* corresponds to the number of clusters in the network, and *X* and *Y* contain the set of communities that appear at different thresholds *d*_*X*_ and *d*_*Y*_ of the Jaccard distance. Following refs 57,58, VI is defined as:


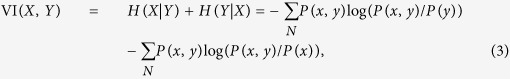


where *H*(*X*|*Y*) is the conditional entropy, the information needed to describe *X* known *Y*. Hence, two partitions *X* and *Y* obtained at different thresholds and that have the same set of communities would result in VI = 0. We scanned VI as a function of the threshold *d* in steps of 0.01. Each threshold provided a partition *X* containing *n* moduli, and constructed as a vector of integers where each value labels the module where a cluster belongs to. For instance, in the example of Fig. 4 a threshold *d* = 0.75 provided *n* = 2 moduli and *X* = {2, 2, 1, 1, 2}. Labels ‘1’ and ‘2’ denote the community index, with clusters {3, 4} shaping the community #1, and clusters {1, 2, 5} the community #2.

For the actual experimental data, we computed VI between a partition at a thershold *d*_*i*_ and all the other partitions at *d* ≠ *d*_*i*_, and computed the average value, MVI_*i*_. Finally, to identify which threshold *d*_*i*_ provided the most significant partition, we plotted the difference ΔMVI = MVI(*d*_*i*+1_) − MVI(*d*_*i*_) for gradually higher *d*_*i*_. A jump in ΔMVI indicated that a small variation in the threshold leaded to a significant change in the structure of communities (see Supplementary Figure 1). Our choice of the optimum threshold *d*_th_ was set as the highest jump in ΔMVI that provided at least two communities.

### Network topological properties

The inferred functional networks are directed and weighted. From their data and following refs 25,59 we calculated using the software package ‘Radatools’^60^ a number of descriptors that provide further details on network traits. The *strength* of node is the sum of all weighted links that either enter or leave a node. The *global efficiency* is the inverse of the characteristic length, which is computed as the mean of geodesic lengths over all couple of nodes. The global efficiency accounts for the information flow across the network, and can be seen as a measure of global network integration. The *local efficiency* is the corresponding measure within neighboring nodes, and reflects the communication performance within neighbors or local subgraphs. The *clustering coefficient* is the fraction of triangles around an individual node, i.e. how likely the neighbors of a node are also neighbors of each other. The higher the clustering coefficient is, the higher the segregation in the network. The *assortativity* is calculated as the correlation between the weighted in– and out–degree of all pairs of linked nodes, and reflects the tendency of a node to connect to others with similar degree. Assortativity is zero in a regular lattice since each pair of linked nodes have the same number of connections, and it is positive in networks formed by modules of different sizes.

### Homogeneous cultures

They were prepared in almost identical conditions as the clustered ones, with the only difference that, prior plating, the PDMS–glass structures were treated overnight with poly-l-lisine (PLL, Sigma). PLL coating favored the anchorage of the neurons on the glass substrate, restricting their motility and effectively giving rise to networks with a homogeneous distribution of neurons on the surface. Culturing, maintenance and actual experiments were performed identically as described for clustered cultures.

For data analysis, and since single neurons were not easily identifiable, ROIs were established as a grid of 30 × 30 elements that covered uniformly each cavity, and each ROI encompassing about 5–10 neurons. We verified that the analysis using groups of neurons instead of single ones did not alter the results, as also observed in other studies with similar cultures^50^.

Homogeneous cultures exhibit a dynamics that is characterized by episodes of collective, highly coherent activity of the entire network (*network bursts*) combined with silent intervals^50^,^61^. This all-or-none behavior excludes the existence of communities. Therefore, the changes in spontaneous activity upon chemical action were quantified as the ratio in the number of bursting episodes after and before the chemical application.

### Statistical significance tests

We used the Student’s t-test for clustered cultures, and the Kolmogorov-Smirnov test to compare homogeneous and clustered networks since the two culture types had different number of experimental realizations. In all the statistical results, the p-value *p* is indicated as * for *p* < 0.05, and ** for *p* < 0.005.

## Additional Information

**How to cite this article**: Teller, S. *et al.* Magnetite-Amyloid-*β* deteriorates activity and functional organization in an *in vitro* model for Alzheimer's disease. *Sci. Rep.* **5**, 17261; doi: 10.1038/srep17261 (2015).

## Supplementary Material

Supplementary Information
