# Supplementary material for: Magnetite-Amyloid-β deteriorates activity and functional organization in an in vitro model for Alzheimer’s disease
Source: Sci Rep. 2015 Nov 26;5:17261. doi: 10.1038/srep17261 (PMC4660300; doi:10.1038/srep17261)
Supplement: Supplementary Information [file srep17261-s1.pdf]

Supplementary information

Magnetite-Amyloid-β deteriorates activity and functional organization in an *in vitro* model for Alzheimer's disease

Sara Teller, Islam Bogachan Tahirbegi, Mònica Mir, Josep Samitier, and Jordi Soriano

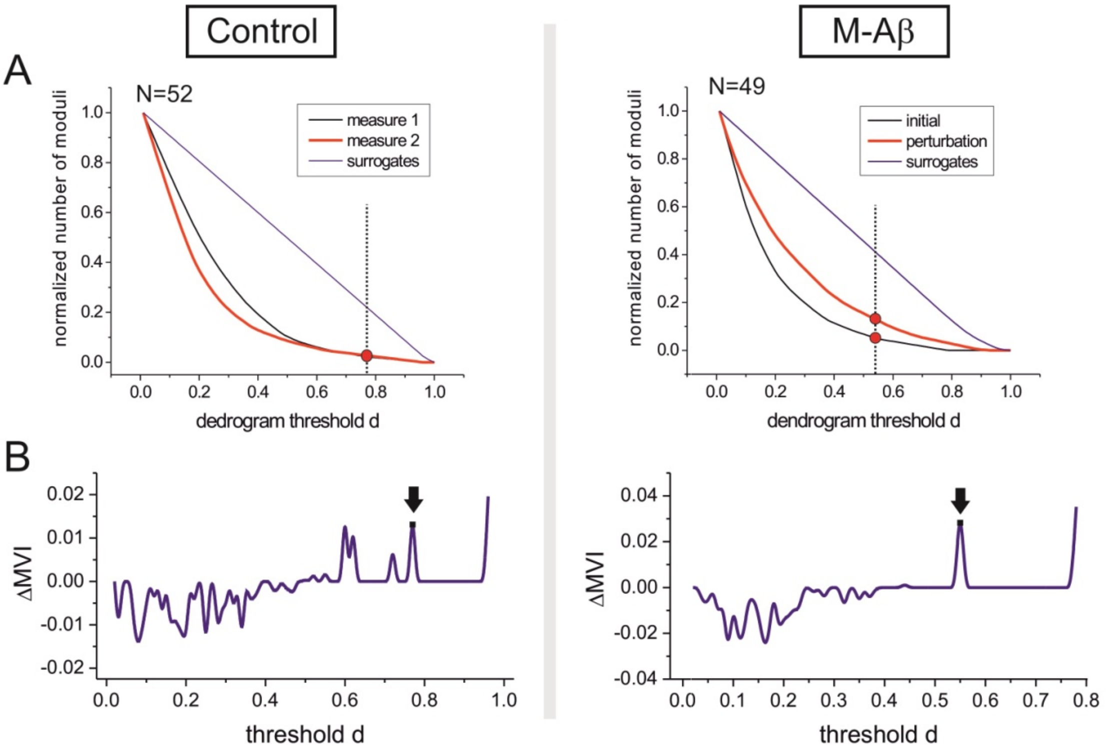

Supplementary Figure 1. Determination of the threshold for the characteristic number of communities. (A) Number of identified moduli, normalized by the number N of clusters in the network, as a function of the threshold d in the dendrogram. For d=0 the number of communities equals the number of clusters. As d grows, clusters with similar history group and the number of communities gradually reduces, until for the extreme case of d=1 all clusters form a single community. The number of communitiies tend to stabilize in between the extreme values for the recorded data. In controls, the distribution of communities between the first and the second measurement slightly varied due to fluctuations in the dynamics of the clusters, with an overall decrease in the number of communities for the shown example. The dashed vertical line and the dot mark the position of the optimum threshold d_{th} (as described next) that defines the characteristic number of communities for the first measurement. Despite fluctuations, the second measurement maintains the number of characteristic communities. For M-Aβ, the perturbation leaded to an overall higher curve. The threshold d_{th} was measured for the initial, unperturbed measurement. Hence, by maintaining the same threshold as reference, the number of communities effectively increased upon perturbation. Data surrogates, which destroy the correlations between clusters, do not show any characteristic number of communities. (B) Determination of the optimum threshold d_{th} using Variation of Information [Karren et al., Phys. Rev. E 77, 1 (2008)]. If X and Y represent, respectively, the set of communities identified in the network at thresholds d_i and d_j, then VI(X,Y)= H(X|Y) + H(Y|X), with H(X|Y) the conditional entropy of X and Y. For the analysis of the data, we first calculated MVI, the average mutual information between a community at threshold d_i and all the other communities at $d \neq d_i$. We then plotted the difference ΔMVI between two consecutive thresholds as a function of d. Big jumps indicated a substantial change in the organization of the communities. The optimum threshold was then set as the value of d that provided the highest jump and with at least two communities (black arrow).

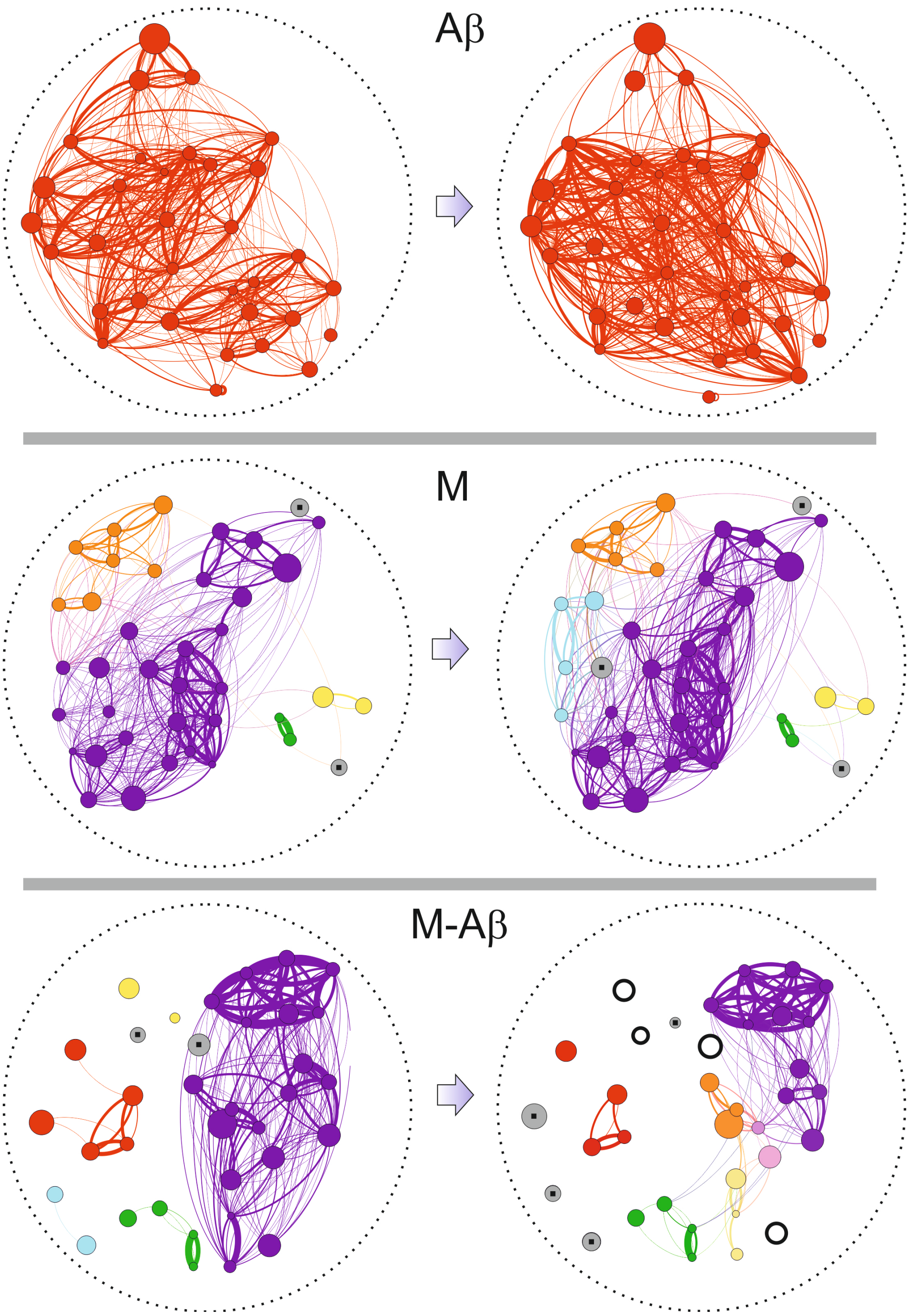

Supplementary Figure 2. Comparison of the functional networks for M, Aβ and M-Aβ. The graphs illustrate the stability of the network moduli and functional connectivity for the M and Aβ cases. Although functional links change in strength and target, the variability is similar to the control measurements. An additional M-Aβ experiment is shown for comparison. Links between clusters correspond to the top functional connections (z-score>1.95, 500 surrogates), with the thickness proportional to the weight of the link. Their direction is not shown for clarity. Clusters and links are color coded according to their participation in a given community, with the thicness of links proportional to their importance. Grey clusters with a square in their center are those that fired independently or that participated equally in different communities. Clusters with thick outlines are those that ceased activity after chemical application.
